# Addressing Sensory Impairment in the ICU: Critically Important for Improving Outcomes

**DOI:** 10.1093/geroni/igaa057.2893

**Published:** 2020-12-16

**Authors:** Lauren Ferrante

**Affiliations:** Yale School of Medicine, New Haven, Connecticut, United States

## Abstract

Millions of older adults are admitted to an intensive care unit (ICU) every year. Many of these patients have preexisting impairment in hearing or vision, but sensory impairment is rarely addressed in the ICU. This talk will present recent work evaluating the association of sensory impairment with functional outcomes among older ICU patients, and discuss barriers to and potential strategies for addressing sensory impairment in the ICU.

